# Mitochondria-Endoplasmic Reticulum Interplay Regulates Exo-Cytosis in Human Neuroblastoma Cells

**DOI:** 10.3390/cells11030514

**Published:** 2022-02-02

**Authors:** Giacomo Dentoni, Luana Naia, Maria Ankarcrona

**Affiliations:** BioClinicum J9:20, Division of Neurogeriatrics, Center for Alzheimer Research, Department of Neurobiology, Care Science and Society, Karolinska Institutet, Visionsgatan 4, 171 64 Solna, Sweden; giacomo.dentoni@ki.se (G.D.); luana.naia@ki.se (L.N.)

**Keywords:** MAM, MERCS, mitochondria, exocytosis, inositol 1,4,5-trisphosphate receptor, MCU

## Abstract

Mitochondria–endoplasmic reticulum (ER) contact sites (MERCS) have been emerging as a multifaceted subcellular region of the cell which affects several physiological and pathological mechanisms. A thus far underexplored aspect of MERCS is their contribution to exocytosis. Here, we set out to understand the role of these contacts in exocytosis and find potential mechanisms linking these structures to vesicle release in human neuroblastoma SH-SY5Y cells. We show that increased mitochondria to ER juxtaposition through Mitofusin 2 (Mfn2) knock-down resulted in a substantial upregulation of the number of MERCS, confirming the role of Mfn2 as a negative regulator of these structures. Furthermore, we report that both vesicle numbers and vesicle protein levels were decreased, while a considerable upregulation in exocytotic events upon cellular depolarization was detected. Interestingly, in Mfn2 knock-down cells, the inhibition of the inositol 1,4,5-trisphosphate receptor (IP3R) and the mitochondrial calcium (Ca^2+^) uniporter (MCU) restored vesicle protein content and attenuated exocytosis. We thus suggest that MERCS could be targeted to prevent increased exocytosis in conditions in which ER to mitochondria proximity is upregulated.

## 1. Introduction

Mitochondria are highly dependent on other organelles and particularly pivotal is their interaction with the endoplasmic reticulum (ER). Mitochondria are closely juxtaposed (10–30 nm) to the ER [1], forming what are known as mitochondria-ER contact sites (MERCS). Five to 20% of the mitochondrial surface is believed to be in contact with the ER [1], and the lipid-raft domain on the ER juxtaposed to mitochondria is referred to as the mitochondria-associated ER membrane (MAM) [2]. Several proteins have been ascribed to this area, having a variety of physiological roles, mainly mediating calcium (Ca^2+^) shuttling between ER and mitochondria [3], bioenergetics [4], ROS production [5], phospholipid exchange [6], mitochondrial fission [7], autophagosome formation [8] and apoptosis [9].

The exact composition of the scaffolds through which the two membranes are connected remains elusive in mammalian cells. It is widely believed that proteinaceous tethers link the two membranes, as protein cleavage results in disassembly of these contacts [1]. Several regulators of MERCS juxtaposition have been identified including PDZ domain-containing protein 8 (PDZD8) [10], vesicle-associated membrane protein-associated protein B (VAPB), and protein tyrosine phosphatase interacting protein-51 (PTPIP51) [11] and Mitofusin 2 (Mfn2) [12]. Mfn2, a crucial protein overseeing mitochondrial fusion [13], was also found to be located in these lipid raft domains, forming homo or heterodimers with Mitofusin 1 (Mfn1) [12]. The exact role of this protein in MERCS remains contested. Mfn2 knock-out MEF cells studies showed decreased interaction between mitochondria and ER-membranes, as well as decreased Ca^2+^-shuttling between ER and mitochondria [12]. These studies were corroborated by observations in cell lines [14,15], brain [16] and neurons [17]. Subsequently, several studies have challenged this view with several independent groups, including ours, reporting that acute Mfn2 knock-down (Mfn2 KD) results in increased interaction between the membranes, assessed through electron micrographs [18,19,20,21], conventional confocal microscopy [20] and with recently characterized fluorescence-based reporters of ER-mitochondria juxtaposition [22,23]. Functionally, acute Mfn2 KD resulted in increased ER to mitochondria Ca^2+^-shuttling [19,20], hence pointing at Mfn2 as a negative regulator of MERCS. These differences are likely due to different cell culture methods used in different laboratories, cell models that react differently or develop distinct compensatory mechanism to loss of Mfn2 and different methods used to assess ER to mitochondria proximity, which have been thoroughly reviewed [24,25].

Ca^2+^-signaling impacts on a variety of cellular mechanisms, including exocytosis [26]. Ca^2+^ entry in neuronal cells is mediated via voltage-gated Ca^2+^ channels triggering release of vesicle cargoes [27]. Vesicle release can also be influenced by the Ca^2+^ buffering capacity of the ER [28,29] or ER-mediated Ca^2+^-release [30,31]. Importantly, ER Ca^2+^-buffering capacity can shape Ca^2+^ signaling interaction with mitochondria, through MERCS. Mitochondria have been shown to modulate Ca^2+^-mediated vesicle release through their Ca^2+^-buffering capacity [32,33,34]; moreover, they are capable of coupling Ca^2+^-buffering and ATP production by activating Ca^2+^-dependent Tricarboxylic acid (TCA) cycle enzymes [35]. Several studies have described the role of mitochondrial bioenergetics in exocytosis, and oxidative phosphorylation (OXPHOS) appears to be of vital importance for the release mechanism, as the inhibition of OXPHOS impaired exocytosis during neuronal activity [36,37]; while on the other hand, enhanced ATP levels induced vesicle release in pancreatic β-cells [38]. Hence, mitochondria act as master regulators of excitability by timely coupling electrochemical signals to metabolic needs. Limited studies have assessed the importance of these interactions between ER and mitochondria in exocytosis. Pharmacological modulation of Ca^2+^-transfer at MERCS can prolong the residual mitochondrial Ca^2+^ and exocytosis [39]. Furthermore, functional modulation of these contacts, through VAPB and PTPIP51 knock-down, has been shown to affect vesicle release [40], although the mechanisms remain elusive. 

Here, we have investigated the role of MERCS in exocytosis in the human neuroblastoma cell line SH-SY5Y, a widely used neuronal model showing neuron-like features [41]. Our data show that Mfn2 downregulation results in increased contacts between the two organelles, a decrease of synaptic-related vesicle protein levels (synaptophysin, synapsin-1), an increase in vesicle-priming SNAP25 target-SNARE proteins, and the upregulation of exocytosis. Interestingly, these effects seem to be dependent on SNAP25 upregulation, as knock-down of SNAP25 increased synaptophysin levels and blocked exocytosis. Furthermore, ER and mitochondrial Ca^2+^-signaling play a vital role in the exocytosis mechanism, as blockage of the inositol 1,4,5-trisphosphate receptor (IP3R) and mitochondrial Ca^2^^+^ uniporter (MCU) activity in increased ER to mitochondria proximity conditions, dampened exocytosis. Hence, we reveal new insights into how the increased interaction of mitochondria and ER can induce changes in exocytosis, and how these abnormalities can be reverted, targeting ER and mitochondrial Ca^2+^ signaling.

## 2. Materials and Methods

### 2.1. Antibodies

The following antibodies were used: Actin (Sigma-Aldrich, St. Louis, MO, USA, #A4700 1:2500), Actin (Sigma-Aldrich, #A5441 1:2500), LC3 (Cell Signaling Technology, Danvers, MA, USA #3868 1:1000), Mfn2 (Abcam, Cambridge, UK, #Ab56889 1:1000), Neuropeptide Y (Cell Signaling Technology, #11976T 1:500), pSer293-PDH (Merck Millipore, Burlington, MA, USA #ABS204 1:1000), PDH (Santa Cruz, Dallas, TX, USA #sc-377,092), SNAP25 (Biolegend, San Diego, CA, USA #850301 1:1000), Synapsin-1 (Cell Signaling Technology, #5297T 1:1000), Synaptophysin (Abcam,#Ab14692 1:1000), Synaptotagmin-1 (Novus Biologicals, Littleton, CO, USA #MAB4364-SP 1:500), Syntaxin-1 (Sigma-Aldrich, #S0664) 1:500), TIM23 (BD Biosciences, Franklin Lakes, NJ, USA #611223 1:1000), and VDAC1 (Abcam, #Ab14734 1:1000). 

### 2.2. Ca^2+^ Imaging

SH-SY5Y cells plated in 96-well plates were washed once with mKRB media (in mM: 140 NaCl, 2.8 KCl, 2 MgCl_2_, 10 HEPES, 2 CaCl_2_, pH 7.4 at 37 °C) and incubated for 45 min, at 37 °C, with 2 mM Fura-2/AM (Invitrogen, Waltham, Massachusetts, USA catalog #F1221), 0.02% pluronic F-127 (Invitrogen, catalog # P3000MP) and 200 mM sulfinpyrazone (Sigma-Aldrich, catalog #S9509-5G) prepared in mKRB. After rinsing with fresh KRB, Fura-2 fluorescence was measured using a FLUOstar OPTIMA spectrofluorometer (BMG Labtech, Ortenberg, Germany) at dual 340/380 nm excitation and 510 nm emission wavelengths. Basal Fura-2 fluorescence was recorded every 20 s for 4 min followed by stimuli with FCCP/oligomycin (2/3.5 μM) (Sigma-Aldrich, catalog #C2920 and #75351) or thapsigargin (2.5 μM) (Sigma-Aldrich, catalog #T9033-.5MG), KCl (50 mM) and 4-Bromo-calcium Ionophore A23187 (2 μM) (Sigma-Aldrich, catalog # B7272). All plotted values were normalized for baseline values. In experiments with thapsigargin, Ca^2+^ was omitted from mKRB.

### 2.3. Cell Culture, Transfections, and Treatments

Neuroblastoma SH-SY5Y cells were obtained from American Type Culture Collection (Manassas, VA, USA) and were cultured in Dulbecco’s Modified Eagle Medium (DMEM) (#41965039, Thermofisher, Waltham, MA, USA) and supplemented with 10 % fetal bovine serum (FBS, #10270098, Thermofisher). siRNA solutions were prepared by mixing Lipofectamine^®^ 2000 Transfection Reagent (#11668019, Thermofisher) with either Mfn2 siRNA mix (SI04375406, SI04342716, SI04217430 and SI04188835, Qiagen, Hilden, Germany), SNAP25 siRNA mix (SI05733308, SI04832128, SI04832114 and SI04832107, Qiagen) or AllStars Negative Control (NC) siRNA (#1027280, Qiagen) in OptiMEM (#31985070, Thermofisher) to a final concentration of siRNA equal to 40 nM. The mix was incubated at room temperature (RT) for 10 min to allow formation of complexes. The mix was added to the plates for 4h, media was substituted to supplemented DMEM and cells were grown for further 72h. Treatment with Xestospongin C (XeC; #1280 Tocris, Bristol, UK) or MCUi4 (#7195, Tocris) 1µM was carried out for 6h before imaging or cell lysis was performed, and control conditions were incubated with an equal volume of DMSO.

### 2.4. Electron Microscopy

Cells were washed twice in PBS (# 14190094, Thermofisher), fixed, and then scraped in 2.5% glutaraldehyde in 0.1 M phosphate buffer. Cell ultrathin sections were prepared using Leica Ultracut UCT (Leica, Wetzlar, Germany) and uranyl acetate and lead citrate were used as contrasting agents. Sections were analyzed through a Tecnai 12 BioTWIN transmission electron microscope (FEI Company, Hillsboro, OR, USA) at 100 kV. Digital images were acquired with a Veleta camera (Olympus Soft imaging Solutions, GmbH, Münster, Germany) at 26,500× magnification. All mitochondria from 10 different cells were imaged per sample. Number of MERCS and mitochondrial profiles, average length of MERCS and mitochondrial perimeter were analyzed using the freehand line tool in ImageJ (NIH, Bethesda, MD, USA). The number of MERCS per mitochondria were obtained by dividing the number of MERCS per number of mitochondrial profiles. The percentage of mitochondria perimeter in contact with ER was analyzed by multiplying the MERCS length per 100 times divided by the mitochondrial perimeter. ≤ 30 nm distance between ER and mitochondria was set as our limit to consider the distance between the two membranes to be considered as contacts. Only images containing vesicles that were clearly distinguishable were analyzed and the whole cellular area was inspected. The number of vesicles was determined by selecting ribosome free, single membranes and rounded vesicles analyzed using the freehand line tool in ImageJ (NIH, USA). Dense core vesicles presented electron dense material, while regular vesicles’ lumen was devoid of electron dense material.

### 2.5. Gene Expression Analysis

An RNeasy Mini Kit (Qiagen, #74104) was used to isolate total cellular RNA, according to manufacturer’s instructions. RNA integrity was confirmed by A260/280 > 1.9. 10 ng of RNA was reversely transcribed into cDNA using the High Capacity cDNA Reverse Transcription kit (Life Technologies, Carlsbad, CA, USA #4368814). Quantitative Real-Time PCR was performed in an Applied Biosystems 7500 fast thermal cycler system (Applied Biosystems, Waltham, MA, USA) using the TaqMan Fast Advanced Master Mix (Applied Biosystems, #4444557) and the following primers: SYN1 (assay ID: Hs00199577_m1), SYP (assay ID: Hs00300531_m1) and ACTB (assay ID: Hs01060665_g1). Analysis of gene expression was performed using the ΔΔCT method and relative gene expression was normalized to actin mRNA levels.

### 2.6. Lactate Dehydrogenase Assay (LDH) Assay

Cytotoxicity in different transfection conditions was assessed through LDH assay (#G1780, Cytotox 96^®^assay, Promega, Madison, WI, USA) according to the manufacturer’s instructions. The LDH absorbance signal was measured at 492 nm with an optical plate reader (FLUOstar galaxy). The maximum LDH release was assessed by incubating cells with 10 µL of Lysis buffer for 30 min prior to analysis. LDH absorbance was normalized to protein concentration, and data is presented as a percentage of NC siRNA.

### 2.7. Oxygen Consumption and Total ATP Levels 

**Figure 1:**
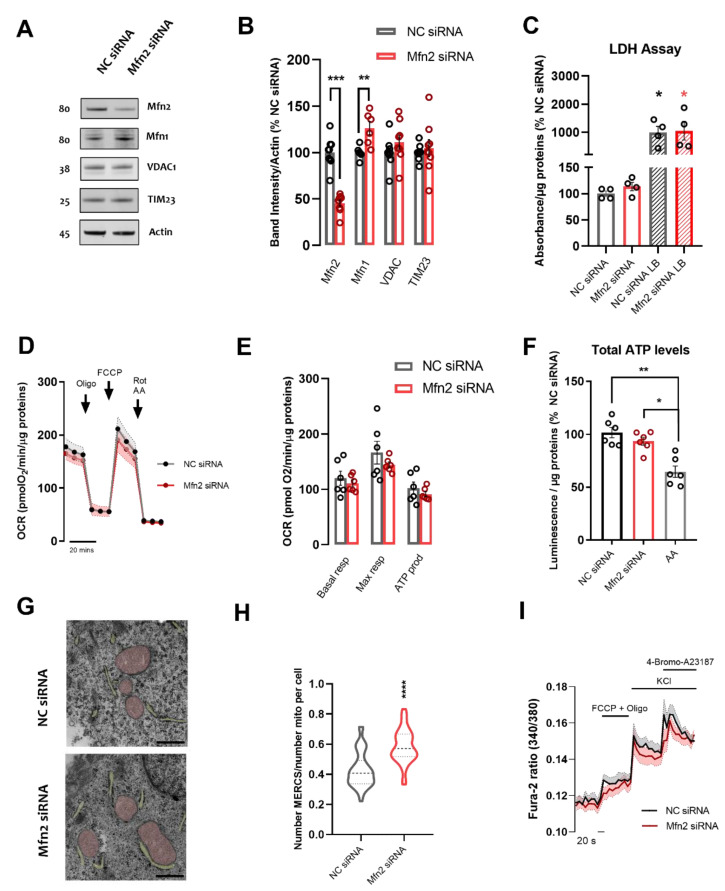
Mfn2 down-regulation increases ER–mitochondria juxtaposition while not affecting cellular nor mitochondrial viability, nor cytoplasmic Ca^2+^ dynamics. (**A**) Representative immunoblots of SH-SY5Y cell homogenate treated with NC siRNA or Mfn2 siRNA. Blots were probed with antibodies against Mfn2, Mfn1, VDAC1, and TIM23, and Actin was used as a loading control. (**B**) Bar graph shows the amounts of protein analyzed once standardized to actin content in each sample (*n* = 9 independent cultures) (**C**) Bar graph shows quantification of LDH assay absorbance normalized to protein content in NC siRNA or Mfn2 siRNA cultures; a Lysis Buffer (LB) was used as a positive control to elicit LDH release (*n* = 4 independent cultures) (**D**) Oxygen consumption rate (OCR) traces showing cellular respiration in NC siRNA or Mfn2 siRNA treated SH-SY5Y cells after the sequential injection of oligomycin (oligo,1 µM), FCCP (1 µM) and Rotenone + Antimycin A (Rot+AA, 0.5 µM) (*n* = 6 independent cultures) (**E**) Bar graphs show quantification of OCR parameters extrapolated from Seahorse XF Cell Mito Stress Test and normalized to protein content, including basal respiration (Basal Resp), maximal respiration (Max Resp) and ATP production (ATP prod) (**F**) Bar graph shows quantification of ATP luminescence normalized to protein content in NC siRNA or Mfn2 siRNA cultures; 5 µM AA was used as a negative control by incubating non treated cells for 1 h prior to analysis (*n* = 6 independent cultures) (**G**) Representative TEM pictures of NC siRNA or Mfn2 siRNA SH-SY5Y cells showing mitochondria (pink) and ER (yellow) in close proximity to each other forming MERCS. Scale bar = 500 nm. (**H**) Violin plot shows quantification of number of MERCS per number of mitochondria per cell (4 independent cultures, 9–10 cells analyzed per culture). (**I**) Quantification of cytosolic Ca^2+^ Fura-2/AM fluorescence transients at baseline and peak amplitude upon different stimuli (*n* = 5–7 independent cultures). Data shown as mean ± SEM. * *p* ≤ 0.05, ** *p* ≤ 0.01, *** *p* ≤ 0.001, **** *p* ≤ 0.0001.

The oxygen consumption rate (OCR) was measured using a Seahorse XFe96 Analyzer (Agilent, Santa Clara, CA, USA). SH-SY5Y cells were grown in Seahorse XF96 microplates (20,000 cells per well) for 72 h post knock-down. SH-SY5Y cells OCR analysis was performed in sodium bicarbonate and phenol red free DMEM (#D5030, Sigma-Aldrich) supplemented with l-glutamine 4 mM, d-glucose 25 mM, pH = 7.4, at 37 °C without CO_2_. Baseline OCR was measured followed by sequential injection of the following drugs: oligomycin 1 μM (#75351, Sigma Aldrich), carbonyl cyanide-4-(trifluoromethoxy) phenylhydrazone 1.5 μM (FCCP, # C2920 Sigma Aldrich), and Antimycin A 0.5 μM (# A8674 Sigma Aldrich) with rotenone 0.5 μM (#R8875, Sigma Aldrich). OCR values were reported according to the Seahorse XF Cell Mito Stress Test (Agilent) and values obtained in pmolO_2_/min were normalized to the total amount of protein. Representative traces are shown in Figure 1D with each point representing the average values of 6 different independent cultures. 

Total ATP levels were measured using the CellTiter-Glo^®^ Luminescent Cell Viability Assay (# G7570, Promega, Madison, WI, USA) according to the manufacturer’s instructions. In the negative control condition, cells were pre-treated for 1 h with 5 µM mitochondrial complex III inhibitor antimycin A. Luminescence was normalized to protein concentration.

### 2.8. SDS-PAGE and Immunoblotting

Cells were lysed in equal volumes of RIPA buffer with 1×proteinase (GBiosciences, St. Louis, MO, USA #786433), 1xphosphatases (Sigma-Aldrich, #P0044) inhibitors, and benzonase solution consisting of: 50 mM Tris, 4 mM MgSO_4_ and 1x benzonase (EDM Millipore, Burlington, MA, USA #70664-10KUN). Homogenates were centrifuged for 10 min at 10,000 rpm and the supernatant was stored at −20 °C. Protein concentration of samples was determined using the Pierce™ BCA Protein Assay (Thermofisher). 15–25 µg of proteins were run on NuPAGE 4–12% Bis-Tris gels (ThermoFisher, #NP0335BOX) and transferred to a nitrocellulose membrane. Membranes were blocked with 5 % powdered milk in TBS-T, then probed overnight at 4 °C with appropriately diluted primary antibody in blocking solution. Blots were washed and incubated with fluorophore-coupled secondary antibodies (Li-Cor Biosciences, Lincoln, Nebraska, USA IRDye^®^ 680RD Donkey anti-Mouse IgG# 926-68072, IRDye^®^ 800CW Donkey anti-Mouse IgG# 926-32212, IRDye^®^ 800CW Donkey anti-Rabbit IgG# 926-32213) for 1 h at room temperature. Proteins were visualized using Odyssey^®^ Infrared Imaging system (Li-Cor Biosciences) and Image Studio Lite 5.2 (Li-Cor Biosciences) was used to analyze band intensity, which were normalized to respective loading control (actin) and data presented as % of NC siRNA.

### 2.9. SypHy Imaging

SH-SY5Y cells were co-transfected with 1 µg of CMV::SypHy A4 construct, a kind gift from Leon Lagnado, University of Sussex (Addgene plasmid # 24478; http://n2t.net/addgene:24478; accessed on 31 January 2022, RRID:Addgene_24478) [42]. After 72 h of transfection, the transfected coverslips were washed and incubated in HBSS (Thermofisher, #14025050) and imaging was performed with a Carl Zeiss LSM800 inverted confocal microscope equipped with a C-Apochromat 40X oil immersion lens using Zen Blue software (Zeiss, Oberkochen, Germany). Temperature was maintained at 37 °C throughout acquisition. Images were acquired at 10 s intervals, and after 1 min of basal recording, KCl (50 mM) was added to the medium to induce depolarization and the release of vesicles. Up to five cells were imaged on each coverslip in at least four independent cultures. Images were analyzed using ImageJ software, time-lapses were background subtracted by using a random background region and the average grey area was determined in a size-consistent circular non-nuclear region of interest (ROI) area of the cell. The fluorescence intensity (F) of each ROI in the various frames was normalized to its respective pre stimulation intensity (F_pre_) and plotted against time. Area under curve and slope of the curve were determined using XY analysis in GraphPad Prism 8.00 (GraphPad, San Diego, California, USA). Ratiometric analysis of final fluorescence to initial fluorescence (F_f_/F_0_) was calculated by dividing the final fluorescence value of ROI by the initial ROI fluorescence for each curve analyzed.

### 2.10. Statistical Analysis

Data was analyzed using GraphPad Prism 8.00 (GraphPad). Pairs of samples were compared by a non-parametric independent test (Mann–Whitney U-test). Multiple sample analysis was performed using a Kruskal–Wallis test, followed by Dunn’s multiple comparison test. Outliers were evaluated using the ROUT (Q = 1%) method and eliminated when existent. All values are expressed as mean ± standard error of the mean (SEM), *n* = corresponds to number of independent experiments or number of individual measurements, * *p* ≤ 0.05, ** *p* ≤ 0.01, *** *p* ≤ 0.001, **** *p* ≤ 0.0001. Values were considered statistically significant when *p* ≤ 0.05.

## 3. Results

### 3.1. Mfn2 Acts as a Negative Regulator of MERCS in SH-SY5Y Cells

To assess whether MERCS could affect vesicle dynamics in SH-SH5Y cells, we decided to knock-down Mfn2 as a well-established method to modulate the juxtaposition between mitochondria and ER. Due to conflicting results obtained using different conditions and cell lines [12,19,20], we decided to focus on the effect of acute Mfn2 knock-down in SH-SY5Y cells.

Firstly, we modulated MERCS by treating cells with either negative control (NC) siRNA or Mfn2 siRNA for 72 h (Figure 1A). Knock-down (KD) of Mfn2 was validated through western blotting, revealing an average decrease of 55% in Mfn2 levels (Figure 1A,B, *p* ≤ 0.001), while neither the outer mitochondrial membrane resident protein VDAC nor the inner mitochondrial membrane resident protein TIM23 levels were affected (Figure 1A,B), hence suggesting that the treatment did not alter overall mitochondrial mass. No differences were detected in LDH-release in either condition (Figure 1C), confirming that Mfn2 KD did not affect overall cell viability. Furthermore, we assessed mitochondrial health by analyzing the oxygen consumption rate and total cellular ATP levels in NC and Mfn2-KD SH-SY5Y cells. There were no significant differences in either the OCR parameters assessed (Figure 1D,E) nor in the total ATP levels (Figure 1F) when Mfn2 was downregulated, compared to NC siRNA conditions.

To analyze MERCS quantitatively, we assessed proximity between the two organelles by transmission electron microscopy (TEM). Proximity between the two membranes closer than 30 nm was considered a contact [1]. We analyzed the length and number of MERCS, normalizing the number of MERCS per number of mitochondria in the cell to account for changes in the number of mitochondria in cells (Figure 1G,H). As previously reported, we found a substantially upregulated interaction between ER and mitochondria. Indeed, we detected an increased number of contacts per mitochondria in Mfn2 siRNA SH-SY5Y cells (Figure 1G,H, 36 % increase compared to NC siRNA, *p* ≤ 0.001). A small ~10% increase in MERCS length was observed; however, overall, no differences were seen in the percentage of mitochondria in contact with ER (Figure S1A,B). Furthermore, as Mfn2 is a master regulator of mitochondrial dynamics [13], we assessed average mitochondria perimeter and number of mitochondria in these cells in order to account for any changes in the mitochondrial network (Figure S1C,D). In accordance with previous studies [19], there were no significant differences detected in mitochondrial number nor perimeter upon Mfn2 KD. This might be explained by partial compensation of mitochondrial fusion through the Mfn2 homologue Mfn1, whose levels were shown to be upregulated upon Mfn2 KD (*p* ≤ 0.01, Figure 1A,B). 

Since MERCS are pivotal players in intracellular Ca^2+^ dynamics, we further assessed basal, mitochondrial and ER [Ca^2+^] using the ratiometric cytoplasmic Ca^2+^-binding dye Fura-2/AM. No differences in cytosolic [Ca^2+^] were detected between NC siRNA and Mfn2 siRNA treated SH-SY5Y cells. Similarly, no differences were detected upon depolarization of cells (50 mM KCl) and mitochondria (FCCP+AA), or by eliciting ER-Ca^2+^ store release (Thapsigargin) (Figure 1I, Figure S1G). Conditions that promote ER to mitochondria apposition encourage the uptake of Ca^2+^ by mitochondria in response to Ca^2+^ release from the ER by establishing Ca^2+^ hot spots at MERCS. The role of Mfn2 KD in increasing Ca^2+^ shuttling from ER to mitochondria has been widely characterized by us and others [19,20]. While we did not directly measure ER to mitochondria Ca^2+^ shuttling in this study, we confirmed increased mitochondrial Ca^2+^ matrix levels indirectly through measurements of the phosphorylated pyruvate dehydrogenase (pPDH) and total pyruvate dehydrogenase (PDH) ratio. PDH is a mitochondrial matrix enzyme that connects glycolysis to the Krebs cycle and is dephosphorylated and activated by Ca^2+^ dependent phosphatases [35]. Here the pSer293-PDH/total PDH ratio decreased in Mfn2 KD cells (Figure S1E,F), suggesting an increased mitochondrial matrix [Ca^2+^] content, regulated by ER to mitochondria Ca^2+^ transfer at MERCS, and leading to dephosphorylation and activation of PDH [43].

Overall, the data show that Mfn2 acts as a negative regulator of contacts. Mfn2 KD does not affect cellular and mitochondrial health, overall mitochondrial morphology nor cytoplasmic [Ca^2+^] under basal and stimulated conditions.

### 3.2. Upregulation of ER to Mitochondria Proximity Depletes Number of Vesicles, Increases SNAP25 Protein Levels and Boosts Exocytosis

Once established that our model resulted in increased MERCS, we set out to test whether modulation of MERCS influences exocytosis. SH-SY5Y cells present a variety of vesicular proteins and two types of secretory vesicles: small vesicle and dense core vesicles [44]. Furthermore, these cells are capable of releasing and synthesizing neurotransmitters [44,45,46] and neuropeptides such as neuropeptide Y [46]. Therefore, we decided to use these cells, as a basic model for studying vesicular release. 

**Figure 2:**
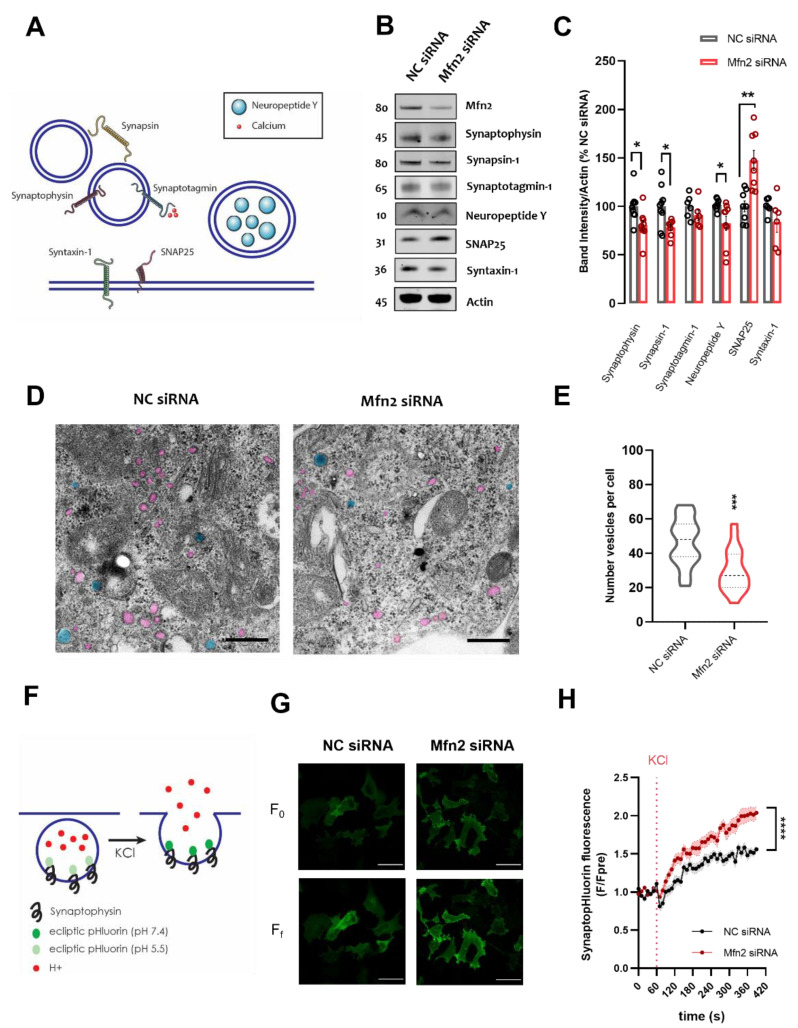
Mfn2 down-regulation decreases vesicular protein levels and vesicle number, while increasing SNARE SNAP25 protein levels and SynaptopHluorin (SypHy) fluorescence. (**A**) Schematic representation of some of the proteins involved in vesicular release mechanisms (**B**) Representative immunoblots of NC siRNA or Mfn2 siRNA SH-SY5Y cell homogenates. Blots were probed with antibodies against Mfn2, Synaptophysin, Synapsin-1, Synaptotagmin-1, Neuropeptide Y, SNAP25, Syntaxin-1 and actin which was used as a loading control (**C**) Bar graph shows the amounts of protein analyzed once standardized to actin content in each sample (*n =* 6–9 independent cultures) (**D**) Representative TEM pictures of NC siRNA or Mfn2 siRNA SH-SY5Y cells showing vesicles (pink) and dense core vesicles (blue). Scale bar = 500 nm. (**E**) Violin plots show quantification of number of vesicles per cell (four independent cultures, five cells analyzed per culture) (**F**) Schematic representation showing the mode of action of SypHy construct upon KCl depolarization (**G**) Representative confocal images of SH-SY5Y cells treated with NC siRNA or Mfn2 siRNA. Panels show initial fluorescence (F_0_) and final fluorescence (F_f_). Scale bar = 10 µm (**H**) Graph shows normalized SypHy fluorescence over time in NC siRNA or Mfn2 siRNA, each timepoint (**F**) was normalized to average pre-stimulation fluorescence levels (F_pre_). After one minute of basal recording, cells were depolarized with 50 mM KCl (*n* = 18–19 cells from 4 independent cultures). The ratiometric difference between F_f_/F_0_ was shown to be significant *p* ≤ 0.0001. * *p* ≤ 0.05, ** *p* ≤ 0.01, *** *p* ≤ 0.001, **** *p* ≤ 0.0001.

Firstly, we carried out a battery of immunoblots of vesicle-related proteins. There are numerous proteins that are involved in vesicle release in the cell and their intracellular location is summarized in Figure 2A. Indeed, we found that the levels of several vesicular proteins and neuropeptides namely synaptophysin, synapsin-1, neuropeptide Y were significantly lower in Mfn2 KD cells compared to NC (Figure 2B,C; *p* < 0.05), while no significant differences were found in synaptotagmin-1 and syntaxin-1 levels (Figure 2B,C). Interestingly, Mfn2 KD cells showed substantial upregulation of SNAP25 (Figure 2B,C, *p* ≤ 0.01), an important Soluble *N*–ethylmaleimide sensitive factor attachment protein receptor (SNARE) protein in the plasma membrane, mediating vesicular docking [26]. As the decrease observed in vesicular protein levels could be caused by Mfn2 mediated changes in protein expression levels, we evaluated human synaptophysin and synapsin-1 mRNA levels in NC and Mfn2 siRNA conditions, through quantitative PCR. No decrease in mRNA levels in either condition was detected (Figure S1H), suggesting that decreased protein levels are not due to reduced transcription.

To further validate our findings on vesicular proteins, we re-analyzed electron micrographs obtained from SH-SY5Y cells to assess the number of vesicles present in our cell model. We were able to observe vesicles devoid of electron dense material in the lumen (highlighted in pink in Figure 2D), as well as dense core vesicles with densely packed material in the lumen (highlighted in blue in Figure 2D), containing neuropeptides such as neuropeptide Y [47]. As predicted and shown in Figure 2D,E, Mfn2 siRNA cells showed a significant downregulation in number of vesicles per cell compared to NC siRNA treated cells (*p* ≤ 0.001).

The decrease in vesicle numbers observed could be associated with downregulation in vesicle-protein production, increased autophagy or increased release mechanisms resulting in depletion of the vesicular pool. We have excluded the first scenario through analysis of mRNA levels (Fig.S1H). Previous studies have reported that downregulation of Mfn2 resulted in reduced autophagosome formation [8,48]. In our study we have observed no changes in the LC3II/LC3I ratio upon Mfn2 siRNA treatment in both basal and Bafilomycin A1 (BAfA1) treated conditions (Figure S1I,J). Hence, we discarded increased autophagy as a possible option for justifying decreased vesicle number in our model and decided to focus on how exocytosis could be affected by Mfn2 KD. Firstly, we tested whether depolarization of SH-SY5Y cells resulted in downregulation of vesicle protein components. Indeed, as previously reported [49], acute simulation by KCl resulted in substantially decreased synaptophysin levels 24h post-stimulation (Figure S4, *p* ≤ 0.05), suggesting that the observed downregulation is due to excessive exocytosis triggered by KCl. Secondly, we co-transfected SH-SY5Y NC siRNA or Mfn2 siRNA cells with SynaptopHluorin A4 plasmid (SypHy). SypHy are generic indicators of synaptic activity and contain a GFP-based pH sensor (pHluorin) linked to a vesicle protein such as synaptophysin-1 [42]. While located inside the acidic lumen of the vesicles, SypHy fluorescence is neutralized; however, by stimulating vesicle release and increasing fusion of vesicles to the plasma membrane, SypHy are exposed to the more neutral extracellular environment, resulting in increased fluorescence [42](summarized in Figure 2F). Hence, increase in SypHy fluorescence enables the visualization of exocytosis events. Mfn2 KD increased SypHy fluorescence upon KCl depolarization over time, suggestive of increased vesicle release (Figure 2G,H). Analysis of traces in Figure 2H established that area under the curve (Figure S2A), slope of the curve (Figure S2B) and ratiometric analysis of final fluorescence to initial fluorescence (Ff/F0; Figure S2C) all resulted in significant upregulation in all parameters analyzed (*p* ≤ 0.001, *p* ≤ 0.01 and *p* ≤ 0.0001 respectively).

The data suggest that increased MERCS through Mfn2 KD affects both vesicular protein levels and the number of vesicles, while increasing target-SNARE SNAP25 levels. Furthermore, upregulation of MERCS through Mfn2 KD substantially increases exocytosis in SH-SY5Y cells.

### 3.3. SNAP25 Downregulation Abrogated Mfn2 KD-Mediated Increased Exocytosis 

As we observed a significant upregulation in SNAP25 levels in Mfn2 KD cells (Figure 2B,C), we decided to investigate possible associations between this target-SNARE and upregulated exocytosis in Mfn2 KD conditions. SNAP25 mediates synaptic vesicle Ca^2+^-dependent fusion by facilitating vesicle apposition to the plasma membrane. Knock-down or knock-out models of this protein result in partial or total inhibition of synaptic transmission and vesicle release in neuronal models [50,51]. Hence, modifying SNAP25 protein levels could be used as an efficient strategy to prevent excessive exocytosis.

**Figure 3:**
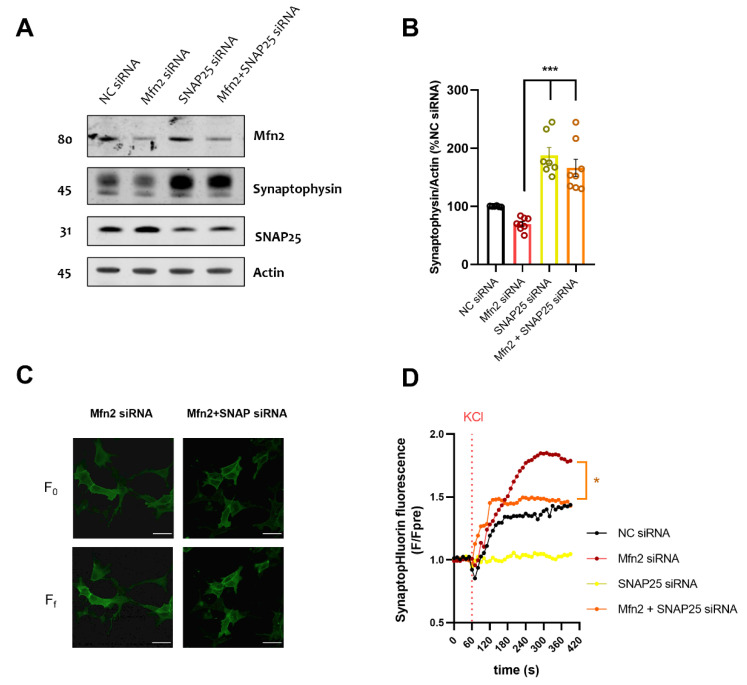
SNAP25 KD rescues Mfn2 KD-mediated vesicle marker downregulation and decreases SypHy fluorescence and exocytosis upon cellular depolarization (**A**) Representative immunoblots of NC siRNA, Mfn2 siRNA, SNAP25 siRNA and Mfn2+SNAP25 siRNA SH-SY5Y cells. Blots were probed with antibodies against Mfn2, synaptophysin and actin which was used as a loading control. (**B**) Bar graph shows the amounts of synaptophysin protein once standardized to actin content in each sample (*n =* 8 independent cultures) (**C**) Representative confocal images of Mfn2 siRNA or Mfn2+SNAP25 siRNA SH-SY5Y cells. Panels show initial fluorescence (F_0_) and final fluorescence (F_f_). Scale bar = 10 µm (**D**) Graph shows normalized SypHy fluorescence over time in NC siRNA, Mfn2 siRNA, SNAP25 siRNA and Mfn2+SNAP25 siRNA, each timepoint (F) was normalized to average pre-stimulation fluorescence levels (F_pre_). After one minute of basal recording, cells were depolarized with 50 mM KCl (*n* = 11–22 cells from 5 independent cultures). The ratiometric difference between Mfn2 siRNA and Mfn2+SNAP25 siRNA F_f_/F_0_ was shown to be significant *p* ≤ 0.05. Data shown as mean ± SEM. * *p* ≤ 0.05, *** *p* ≤ 0.001.

We used a double knock-down approach to decrease Mfn2 and SNAP25 protein levels in the same cells. We reported the successful knock-down of SNAP25 alone (SNAP25 siRNA) and in combination with Mfn2 (Mfn2+SNAP25 siRNA), accounting for a reduction of SNAP25 levels of around 50% compared to NC siRNA conditions. Using immunoblotting we assessed the vesicle marker synaptophysin, as in the previous section. SNAP25 KD alone or in combination with Mfn2 KD led to a significant upregulation of synaptophysin levels compared to Mfn2 siRNA cells (see Figure 3A,B (*p* ≤ 0.01)), indicative of vesicle accumulation. 

To validate our biochemical observations, we co-transfected SH-SY5Y cells with SypHy plasmid with target siRNAs. As previously observed, Mfn2 KD increased SypHy fluorescence after KCl depolarization compared to NC siRNA conditions, indicating upregulated exocytosis (*p* ≤ 0.05, Figure S3A). SNAP25 KD alone led to cells being unresponsive to KCl stimulation, reiterating the importance of this protein in the exocytosis mechanism in neuronal cells (Figure 3C,D, Figure S3A–C). Interestingly, double knock-down of Mfn2 + SNAP25 normalized SypHy fluorescence to NC siRNA levels (*p* ≤ 0.05, Figure 3C,D), suggesting that SNAP25 KD can decrease but not abolish Mfn2 KD-mediated exocytosis. The slope of the curve (Figure S3B) and ratiometric analysis of final fluorescence to initial fluorescence (F_f_/F_0_; Figure S3A) revealed significant upregulation (*p* ≤ 0.05 and *p* ≤ 0.01 respectively) between Mfn2 KD and Mfn2+SNAP25 KD conditions, while area under curve remained unchanged between these two conditions (Figure S3C). 

### 3.4. Ca^2+^-Shuttling between ER and Mitochondria Is Important in Modulating Vesicle Release Mechanisms

**Figure 4:**
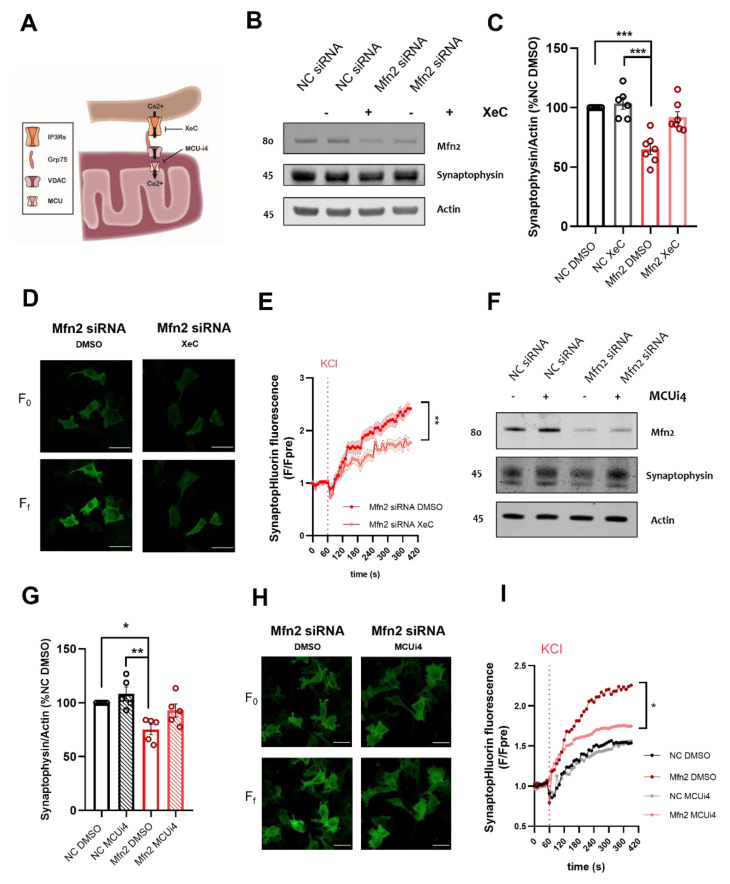
Inhibition of ER to mitochondria Ca^2+^ shuttling abrogates Mfn2-dependent down-regulation of synaptophysin levels and increased exocytosis (**A**) Schematic representation showing mode of action of Xestospongin C (XeC) and MCUi4 at MERCS (**B**) Representative immunoblots of NC siRNA or Mfn2 siRNA SH-SY5Y cells treated or 6 h with DMSO or 1 µM XeC. Blots were probed with antibodies against Mfn2, synaptophysin and actin, which was used as a loading control. (**C**) Bar graph shows the amounts of synaptophysin protein once standardized to actin content in each sample (*n =* 5 independent cultures) (**D**) Representative confocal images of Mfn2 siRNA SH-SY5Y cells treated with DMSO or XeC 1 µM for 6 h. Panels show initial fluorescence (F_0_) and final fluorescence (F_f_). Scale bar = 10 µm (**E**) Graph shows normalized SypHy fluorescence over time in Mfn2 siRNA treated with DMSO or XeC, each timepoint (**F**) was normalized to average pre stimulation fluorescence levels (F_pre_). After one minute of basal recording, cells were depolarized with 50 mM KCl (*n* = 24–27 cells from four independent cultures). Ratiometric difference between F_f_/F_0_ was shown to be significant *p* ≤ 0.01. (**F**) Representative immunoblots of NC siRNA or Mfn2 siRNA SH-SY5Y cells treated for 6h with DMSO or 1 µM MCUi4. Blots were probed with antibodies against Mfn2, synaptophysin and actin which was used as a loading control. (**G**) Bar graph shows the amounts of synaptophysin protein once standardized to actin content in each sample (*n =* 5 independent cultures). (**H**) Representative confocal images of Mfn2 siRNA SH-SY5Y cells treated with DMSO or MCUi41 µM for 6 h. Panels show initial fluorescence (F_0_) and final fluorescence (F_f_). Scale bar = 10 µm **I)** Graph shows normalized SypHy fluorescence over time in NC and Mfn2 siRNA treated with DMSO or MCUi4, each timepoint (**F**) was normalized to average pre stimulation fluorescence levels (F_pre_). After one minute of basal recording, cells were depolarized with 50 mM KCl (*n =* 16–19 cells from 4 independent cultures). Ratiometric difference between F_f_/F_0_ was shown to be significant *p* ≤ 0.05 between Mfn2 DMSO and Mfn2 MCUi4. * *p* ≤ 0.05, ** *p* ≤ 0.01, *** *p* ≤ 0.001. Data shown as mean  ±  SEM.

One of the most characterized functions of MERCS in cellular physiology is their role in Ca^2+^ -shuttling between ER to mitochondria [27]. This is achieved through the functional tethering complex at MERCS composed by IP3Rs in the ER, VDAC1 in the outer mitochondrial membrane and GRP75 a chaperone mediating their juxtaposition [52]. On the inner mitochondrial membrane, MCU complex mediates concentration dependent Ca^2+^ import into the matrix [53] (Figure 4A). Several studies have shown that Mfn2 knock-down increases ER to mitochondria Ca^2+^ shuttling in HEK, MEF and SH-SH5Y cells [19,20].

As Mfn2 knock-down increases ER to mitochondria juxtaposition, we decided to abolish Ca^2+^-shuttling between the two organelles by incubating our cells with XeC, a cell-permeable and potent IP3R inhibitor (Figure 4A) [54]. This approach has been used by us and others to assess MERCS related Ca^2+^-mechanisms [55,56]. Firstly, we decided to tackle the substantial loss in vesicle proteins observed in Mfn2 KD conditions; this, as previously discussed, is likely due to vesicle pool depletion caused by increased vesicle release. Through immunoblot analysis we assessed the levels of synaptophysin in NC and Mfn2 siRNA samples treated with XeC or DMSO. Interestingly, we saw that while XeC incubation alone did not alter synaptophysin protein levels in NC DMSO treated cells, in Mfn2 KD treated cells, XeC normalized synaptophysin levels (Figure 4B,C). To further confirm our findings, we assessed SypHy fluorescence in Mfn2 KD SH-SY5Y cells treated with DMSO or XeC. Interestingly, compared to DMSO-treated conditions XeC decreased the amount of SypHy fluorescence upon KCl treatment, resulting in substantial downregulation of exocytosis in Mfn2 KD cells (F_f_/F_0_ *p* ≤ 0.01, Figure 4 D,E, Figure S2D–F).

To further assess the role of Ca^2+^ in modulating the exocytosis at the ER-mitochondria interface, we pharmacologically blocked Ca^2+^ entry through the MCU channel in the inner mitochondrial membrane. In a recent high throughput screen, MCU-i4 has been identified as an inhibitor of MICU1, a crucial component of the MCU complex that regulates channel gating, thus blocking Ca^2+^ entry into the matrix [57]. Similarly, to the experiments carried out with XeC, synaptophysin levels were measured in NC and Mfn2 siRNA cells treated with MCUi4 or DMSO. Like XeC, MCUi4 treatment alone had no effect on synaptophysin protein levels in the NC siRNA samples; while in Mfn2 KD cells, MCUi4 reversed the significant downregulation of synaptophysin reported in DMSO-treated Mfn2 siRNA cells (Figure 4F,G). Exocytosis evaluation further revealed that no changes in SypHy fluorescence were observed in NC siRNA cells treated with MCUi4 compared to DMSO treated NC siRNA cells. However, following KCl treatment, MCUi4 reduced the SypHy fluorescence ratio and slope of the curve (*p* ≤ 0.05 and *p* ≤ 0.001 respectively, Figure 4H,I, Figure S3D–F).

Our observations suggest a novel mechanism through which mitochondria and ER, when closely juxtaposed, modulate vesicle protein levels and exocytosis through IP3R and MCU activity.

## 4. Discussion

MERCS are important subcellular areas that are gaining growing interest in the scientific community. Several physiological functions have been attributed to these structures including Ca^2+^-shuttling, phospholipid and cholesterol synthesis, bioenergetics, and apoptosis [58]. Recently, the idea that MERCS may be important for exocytosis and neuronal communication has been gaining attention (reviewed in [59,60]). However, the mechanisms and the mediators through which these subcellular regions affect vesicle release remain unclear. 

In this study, we measured exocytosis and vesicle number in a neuronal cell line model upon modulation of ER to mitochondria juxtaposition, through Mfn2 KD, to assert how modulation of MERCS affects this physiological function. Through a combination of western blotting, electron microscopy, functional vesicle release readouts and pharmacological interventions, we show that increased juxtaposition of ER and mitochondria leads to increased exocytosis with resulting downregulation in vesicle number and reduction in vesicular proteins dependent on a MERCS-governed Ca^2+^-dependent mechanism.

To our knowledge, two studies thus far have assessed how MERCS affect exocytosis in neuronal cells. One early study in the field, through pharmacological manipulation of ER and mitochondria juxtaposition, suggested that proximity between the membranes influenced vesicle release [39]. Uncoupling of the mitochondria and ER vesicles in presynaptic terminals via nocodazole, a microtubule polymerization disrupting agent, substantially decreased peak exocytosis, assessed through FM 1-43 dye fluorescence. On the other hand, enhanced Ca^2+^-exchange between the organelles with Taxol, a microtubule stabilizer, resulted in upregulated FM 1-43 dye fluorescence [39]. One limitation of this study is that the pharmacological agents used might have MERCS independent and unspecific effects, as they both act on microtubules, which are important structures for vesicle mobilization, anchoring and release [61,62,63]. A more direct method of modulation of these contacts was presented in a recent study where MERCS tethering proteins VAPB and PTPIP51 were knocked-down in rat neuronal cultures. Decrease of either protein resulted in ER to mitochondria disengagement along with impaired exocytosis [40]. Both studies are in accordance with our observations, further confirming that modulation of MERCS affects vesicular release. It is also interesting to note that upon depolarization and neuronal stimulation, MERCS number increases [40]. Hence, from our data, it is tempting to speculate that there might be a direct relationship between depolarization and MERCS-mediated vesicle release. Additionally, supporting these observations, we report increased SNAP25 levels in Mfn2 KD SH-SY5Y cells. SNAP25, as previously mentioned, is a target-SNARE protein participating along with target-SNARE syntaxin-1 and vesicle-SNARE synaptobrevin in the regulation of vesicle exocytosis in neurosecretory cells [26]. Proteolytic cleavage and knock-down of this protein abolish exocytosis and neurotransmitter release [50,51,64]. Furthermore, SNAP25 in chromaffin cells was shown to maintain functional vesicle pools, priming, docking of vesicles as well as fast Ca^2+^-triggered release [65,66]. Taken together, these studies strongly support that upregulated SNAP25 levels in our model may sustain and increase vesicle release. Indeed, upon knock-down of this protein, we saw a substantial upregulation of synaptophysin levels and downregulation of vesicle release, suggesting that modulating SNAP25 levels can control excessive exocytosis. SNAP25 KD blocked increased vesicle release in Mfn2 KD cells, leading to synaptophysin accumulation (Figure 3A,B). The pathways mechanistically connecting Mfn2 ablation and increased MERCS and SNAP25 upregulation are currently unknown. In fact, while SNAP25 KD decreased exocytosis in Mfn2 KD conditions, Mfn2 KD itself recovers SNAP25-mediated decrease in exocytosis (Figure 3). Therefore, Mfn2 and increased MERCS could rescue conditions in which exocytosis is impaired, while ablation of SNAP25 could be used for opposite outcomes. While some studies have investigated the role of SNAP25 and its homologues in plasma membrane Ca^2+^ dynamics [67] and their role in cholesterol delivery to mitochondria [68], many questions remain to be answered regarding the reciprocal relationship of SNAP25, Mfn2, MERCS and mitochondria. The role of SNAP25 in intracellular Ca^2+^ signaling and how MERCS and mitochondrial activity can influence the assembly and function of SNAREs complexes remain currently unknown. Future research should look at these under-investigated aspects of cellular physiology.

Aside from these functional readouts, we also found substantial downregulation of vesicle components in Mfn2 KD SH SY5Y cells, such as synaptophysin and synapsin-1 along with releasable factors such as neuropeptide Y. This data was corroborated by electron microscopy analysis revealing substantial depletion in the vesicular pool in Mfn2 KD cells. These results are consistent with previously reported data obtained in Mfn2 KD hiPSC-induced neurons, which showed decreased synaptophysin clusters, while Mfn2 overexpression had the opposite effect [69]. Although at first glance the decrease in synaptic vesicle number may appear as a conflicting result to the previously reported increase in exocytosis, it is well established that synaptic depression results in a decreased vesicular pool and decrease in synaptic strength with repeated stimulation [70]. Furthermore, brief cell depolarization with KCl has been reported to result in decreased vesicle immunostaining [49], as shown in SH-SH5Y cells (Figure S4A,B).

The structural and functional juxtaposition between mitochondria and ER impacts on Ca^2+^-signaling, modulating cell bioenergetics and physiological processes. Blocking ER to mitochondria Ca^2+^-shuttling, via Xestospongin B treatment, deeply impairs mitochondrial ATP synthesis [71]. Similarly, in endocrine cells, MCU downregulation reduced matrix Ca^2+^ increases, mitochondrial activity, and insulin release. [72]. On the other hand, increased Ca^2+^-shuttling such as in the early phases of ER stress [73] or during neuronal depolarization [40], may be used by the cell to provide enough energy to carry out their functions properly. Increased dephosphorylation of pSer293-PDH in Mfn2 KD SH-SY5Y cells (Figure S1E,F), supports the idea of increased ER to mitochondria Ca^2+^-shuttling in our model. As exocytosis requires both ATP and Ca^2+^-signaling, it was not surprising to find that increasing mitochondria to ER juxtaposition through Mfn2 KD altered vesicle release. Indeed, this function, associated with mediating Ca^2+^-shuttling between ER and mitochondria at MERCS, was confirmed by the IP3R inhibitor XeC and MCU inhibitor MCUi4 selectively abrogating the effect of Mfn2 knock-down on exocytosis. Interestingly, this effect was not observed in conditions where normal ER to mitochondria apposition was observed, with XeC and MCUi4 having a negligible effect on vesicle marker synaptophysin accumulation and exocytosis (Figure 4). Although neither effect on mitochondrially derived nor total ATP upon Mfn2 knock-down were detected in this or other studies (Figure 1D–F) [19,20], these changes may be masked by increased ATP demand due to enhanced vesicular release and lead to seemingly unaltered ATP levels. Future studies should investigate the full metabolic profile of Mfn2 knock-down cells to resolve this inconsistency.

Mitochondria Ca^2+^-buffering, after a depolarizing stimulus, has been shown to increase the probability of vesicle release, due to gradual discharge of this ion [32,74]; hence, upregulated ER to mitochondria communication could promote this mechanism. While in the short term Ca^2+^-shuttling to mitochondria can have positive effects on cellular function, on the other hand sustained high levels of this ion in the mitochondrial matrix can lead to mitochondrial depolarization, impaired oxidative phosphorylation, mitochondrial swelling, and outer mitochondrial membrane permeabilization, resulting in damaged mitochondria incapable of buffering cytosolic Ca^2+^ [75], and eventually promote apoptosis [58]. Indeed, defective mitochondrial presynaptic Ca^2+^-buffering [76] or lack of presynaptic mitochondria [77] resulted in a substantial increase in cytosolic Ca^2+^ and vesicle release upon depolarization. Similarly, mitochondrial damage caused by sustained Ca^2+^-shuttling from the ER may lead to uncontrolled and sustained exocytosis. While no damage in mitochondrial health was detected in the present study, and no overall upregulation in cytosolic [Ca^2+^] was observed in this and other studies (Figure 1I) [19,20], plasma membrane channels, ER and mitochondria could optimize Ca^2+^-signals elevation in the cytoplasm generating micro-domains by mediating local upregulation of the Ca^2+^-signal within the sites of release [78,79,80,81]. This would allow for the local upregulation of the Ca^2+^-signal at exocytosis sites and could inhibit Ca^2+^-mediated toxicity. Furthermore, Mfn2 modulation could affect other MERCS functions that have not been assessed in this study, such as phospholipid, cholesterol exchange and ROS production, which all have important roles in vesicle release [82,83,84].

## 5. Conclusions

We report that the increased juxtaposition between ER to mitochondria results in increased exocytosis, blocked by MCUi4 and XeC sensitive Ca^2+^-shuttling at MERCS. While this study was carried out in neuroblastoma cells, it is highly possible that a similar mechanism could be observed in neurons, and future studies should address the importance of Ca^2+^-shuttling at MERCS in presynaptic terminals. This study has elucidated a novel mechanism mediated at MERCS, opening possibilities for new interventions to tackle increased MERCS and vesicular dysfunction.

## Data Availability

The data presented in this study are available on request from the corresponding author.

## Supplementary Materials

The following are available online at https://www.mdpi.com/article/10.3390/cells11030514/s1, Figure S1: Analysis of MERCS, Ca^2+^ imaging and dynamics, mRNA synaptic protein levels and autophagy in NC and Mfn2 siRNA conditions. Figure S2: Further quantification of SypHy curves in Figure 2H and Figure 4E. Figure S3: Further quantification of SypHy curves in Figure 3D and Figure 4I. Figure S4: Synaptophysin immunoblots of control SH-SY5Y cells or cells treated with KCl 50 mM stimulation.
