# Supplementary material for: Mitochondria-Endoplasmic Reticulum Interplay Regulates Exo-Cytosis in Human Neuroblastoma Cells
Source: Cells. 2022 Feb 2;11(3):514. doi: 10.3390/cells11030514 (PMC8834387; doi:10.3390/cells11030514)
Supplement: Supplementary file 1 [file cells-11-00514-s001.zip › cells-1505158-supplementary.pdf]

Supplementary Figure 1

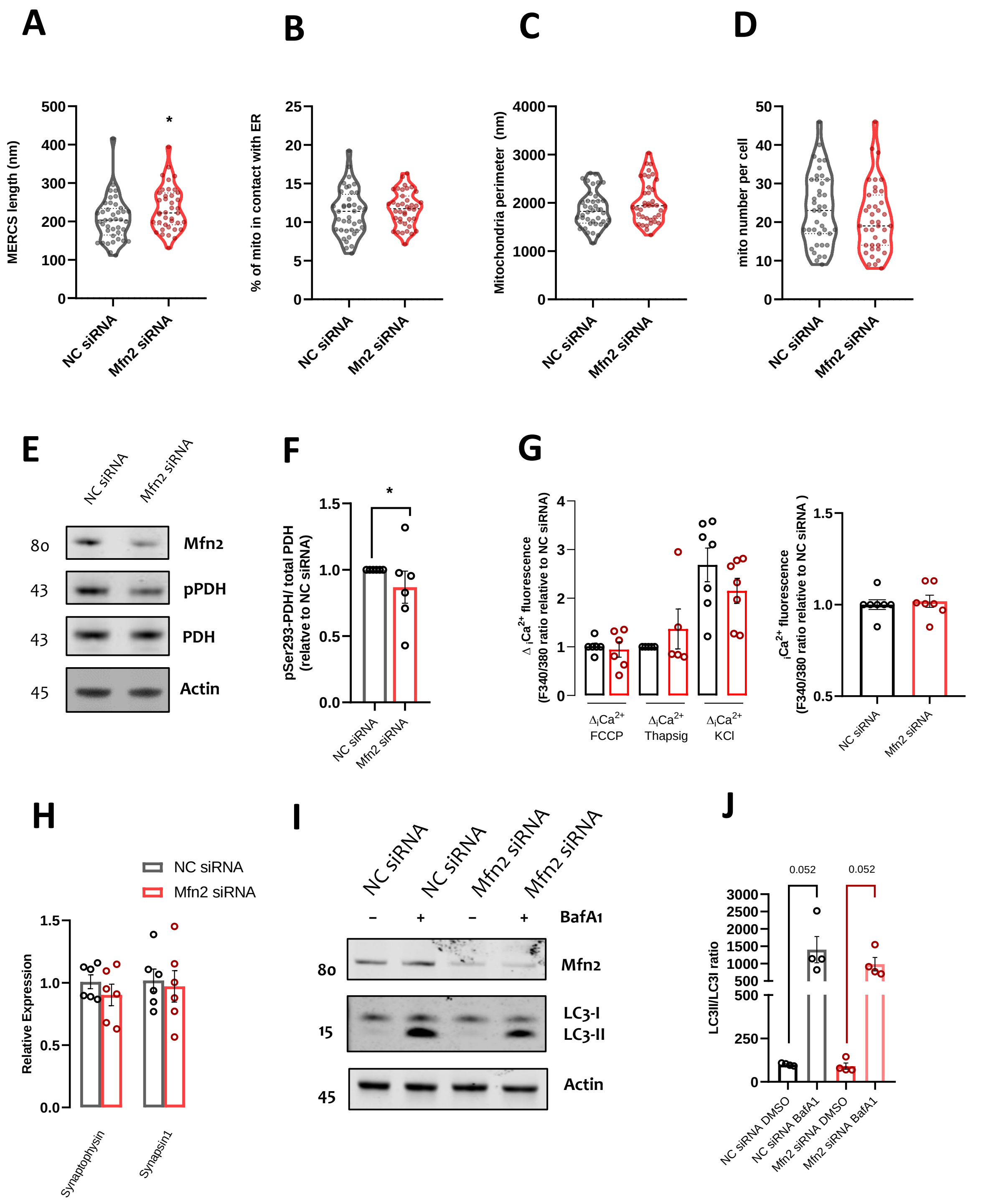

Supplementary Figure 1 **Analysis of MERCS, Ca2+ imaging and dynamics, mRNA synaptic protein levels and autophagy in NC and Mfn2 siRNA conditions** **A)** Further analysis of TEM figures shown in Fig.1A of NC siRNA and Mfn2 siRNA SH-SY5Y cells. Violin graphs show quantification of MERCS length **B)** % of mitochondria in contact with ER **C)** average mitochondrial perimeter per cell and **D)** number of mitochondria per cell. (*n* = 4 independent cultures, 9-10 cells analyzed per culture). **E)** Representative immunoblots of NC siRNA or Mfn2 siRNA SH-SY5Y cells. Blots were probed with antibodies against Mfn2, pSer293-PDH, total PDH and actin which was used as a loading control. **F)** Bar graph shows ratio pSer293-PDH and total PDH protein levels (*n* = 5 independent cultures). **G)** Quantifications of Ca^{2+} traces in Figure 1H **H)** Graph shows the quantification of gene expression analysis of vesicle-related proteins synaptophysin and synapsin-1 by qPCR (*n* = 6 independent cultures). **I)** Representative immunoblots of NC siRNA or Mfn2 siRNA SH-SY5Y cell treated with 50 nM Bafilomycin A1 (BafA1) or non-treated for 5h to block autophagy. **J)** Bar graph shows ratio LC3II to LC3I protein levels (*n* = 4 independent cultures). Data shown as mean ± SEM. * $p \leq 0.05$

Supplementary Figure 2

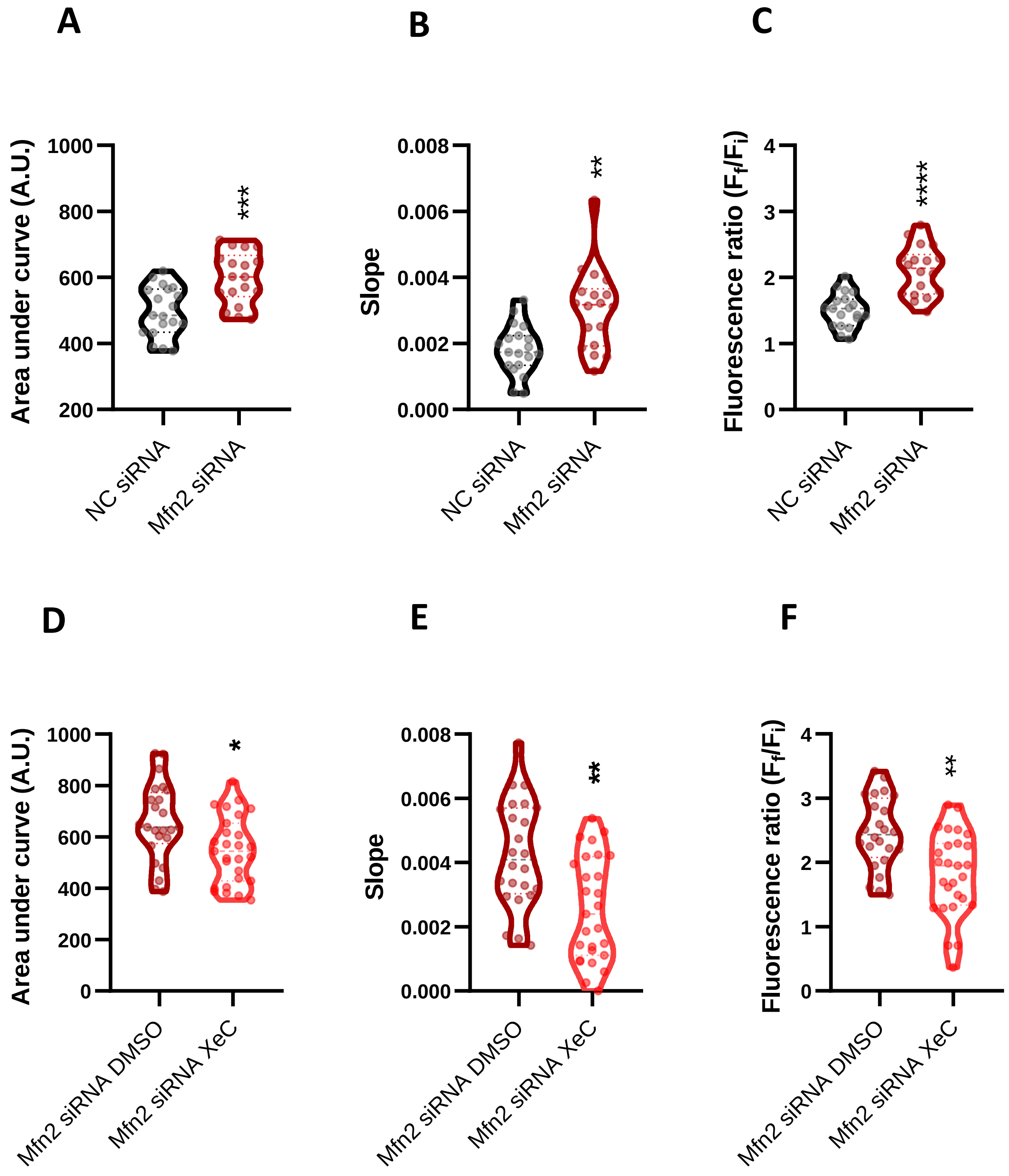

<u>Supplementary Figure 2</u> **Further quantification of SypHy curves in Figure 2H and Figure 4E A)** Violin plots show quantification of curve and data in Figure 2 G-H, area under the curve **B)** slope of curve and **C)** ratiometric analysis of F_f/F_0. **D)** Violin plots show quantification of curve and data in Figure 4D-E area under the curve **E)** slope of curve and **F)** ratiometric analysis of F_f/F_0. * $p \leq 0.05$, ** $p \leq 0.01$

Supplementary Figure 3

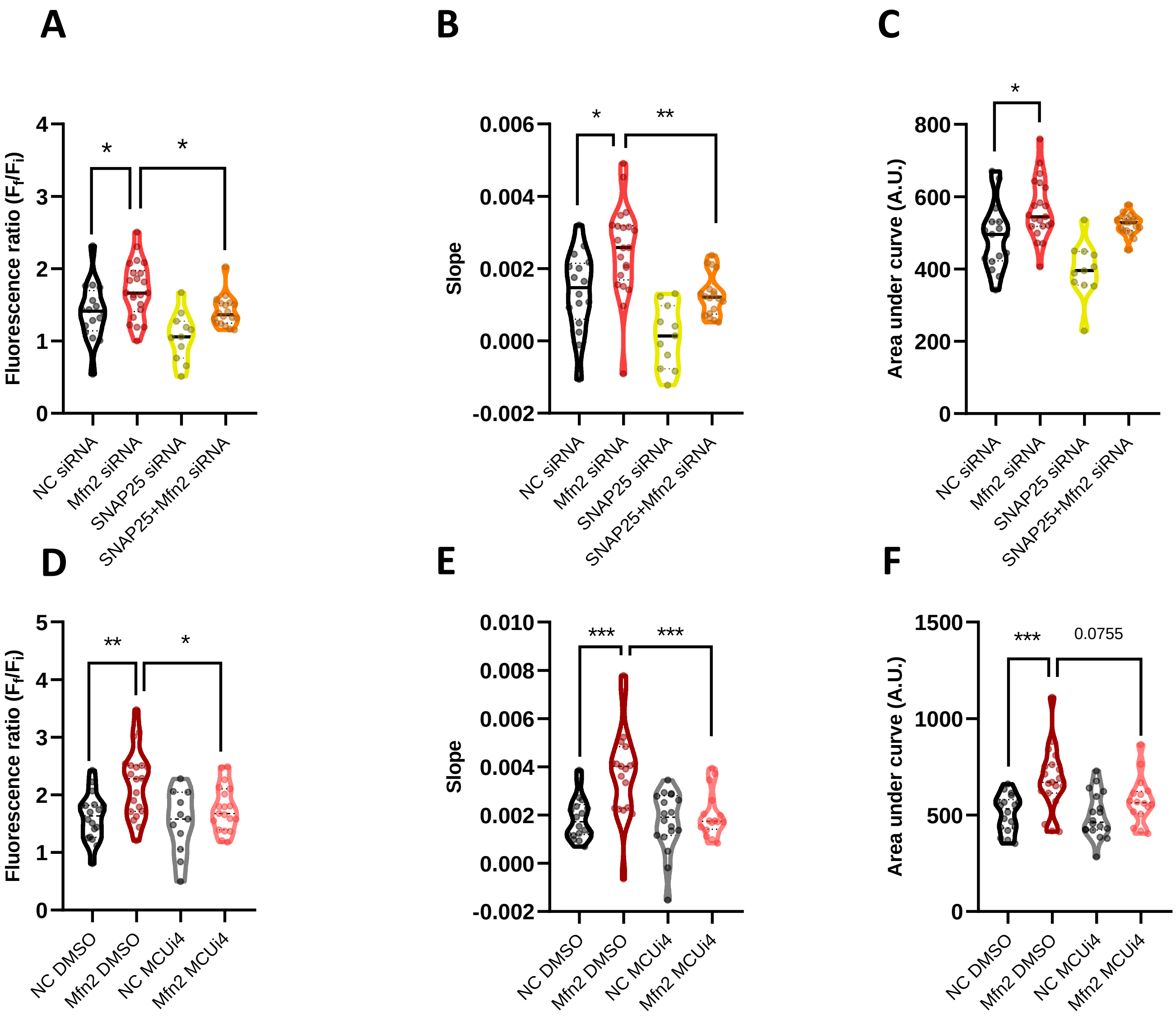

Supplementary Figure 3 Further quantification of SypHy curves in Figure 3D and Figure 4I. Violin plots show quantification of curve and data in Fig.3D **A)** ratiometric analysis of F_f/F_0 **B)** slope of curve and **C)** area under the curve. Violin plots show quantification of curve and data in Fig.4I **D)** ratiometric analysis of F_f/F_0 **E)** slope of curve and **F)** area under the curve. * $p \leq 0.05$, ** $p \leq 0.01$

Supplementary Figure 4

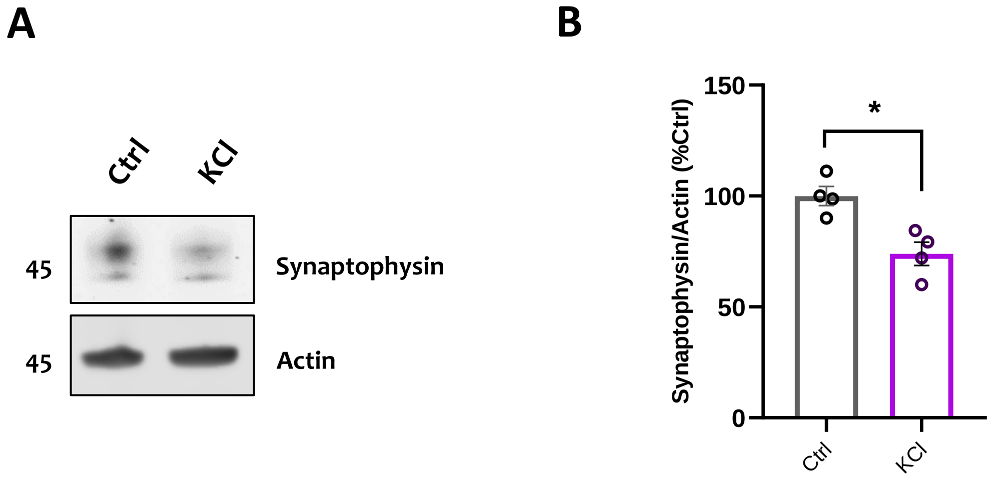

<u>Supplementary Figure 4</u> **Synaptophysin immunoblots of control SH-SY5Y cells or cells treated with KCl 50 mM stimulation A)** Representative immunoblots of control SH-SY5Y cells or cells treated with brief KCl 50 mM 15 mins stimulation and lysed 24h later. Blots were probed with antibodies against Synaptophysin and actin which was used as a loading control. **B)** Bar graph shows the amounts of synaptophysin analyzed once standardized to actin content in each sample (n = 4 independent cultures) Data shown as mean ± SEM.* $p \leq 0.05$.
